# Inadequate Gestational Weight Gain and Associated Factors Among Pregnant Women in Gamo Zone Public Hospitals, South Ethiopia: A Facility‐Based Cross‐Sectional Study

**DOI:** 10.1155/jp/7453325

**Published:** 2026-03-18

**Authors:** Simegn Wagaye Kefene, Fasika Merid, Rahel Hailu, Rahel Abera Alula, Selamnesh Tesfaye, Tamirat Gezahegn Guyo

**Affiliations:** ^1^ Department of Public Health, Arba Minch College of Health Sciences, Arba Minch, Ethiopia, amu.edu.et; ^2^ Regional Data Management Center, South Ethiopia Region Health Bureau Public Health Institute, Jinka, Ethiopia

**Keywords:** inadequate gestational weight gain, public hospitals, South Ethiopia

## Abstract

**Background:**

Globally, inadequate gestational weight gain is a significant public health problem. It may lead to poor pregnancy outcomes. It is associated with the development of a small for gestational age fetus, prematurity, and low birthweight. It is grave trouble in middle‐ and low‐income countries like Ethiopia. However, evidence is scarce on the magnitude of inadequate gestational weight gain and associated factors in our country, particularly in the study area. Hence, this study is aimed at assessing inadequate gestational weight gain and associated factors among pregnant women in Gamo zone public hospitals in South Ethiopia.

**Methods:**

A facility‐based cross‐sectional study was conducted from February 01, 2024, to March 30, 2024, among systematically selected 373 pregnant women. The data were collected by using a structured interviewer‐administered questionnaire, patient record review, and physical measurements. A binary logistic regression model was used to assess the association between dependent and independent variables. A *p* value < 0.05 with its 95% confidence interval (CI) was considered statistically significant and interpreted accordingly.

**Results:**

The magnitudes of inadequate gestational weight gain were 53.1% (95% CI: 48%, 58.4%). First ANC visit after 8 weeks of gestational age (adjusted odds ratio (AOR) = 1.79; 95% CI: 1.02, 3.14), unable to read and write (AOR = 2.86; 95% CI: 1.37, 5.98), and primigravidity (AOR = 2.86; 95% CI: 1.37, 5.98) were the significant predictors of inadequate gestational weight gain.

**Conclusion:**

The findings of this study revealed that more than half of pregnant women still gain inadequate weight, and it has complex relationships with various factors, including women′s education, primigravidity, and gestational age at first ANC contact. Therefore, a comprehensive approach is needed that considers the interplay of these various factors to effectively address the issue of inadequate gestational weight gain.

## 1. Introduction

Gestational weight gain (GWG) refers to the weight gain from pregnancy to birth to support adequate fetal growth and development and breastfeeding after birth [1]. The amount of weight gain is determined in relation to and depending on the existing maternal preconception body mass index (BMI), of which the cutoff values are categorized by the World Health Organization (WHO) [2]. In addition, the Institute of Medicine (IOM) recommends that underweight, normal, overweight, and obese women gain 12.5–18 kg, 7–11.5 kg, and 5–9 kg during pregnancy, respectively. Therefore, as per the recommendations of IOM, inadequate GWG is defined as weight gain below the recommended values in all BMI categories of pregnant women starting from conception up to birth [3].

The global prevalence of inadequate GWG, which is below the 2009 IOM recommendation, is shown to be 39.4% [4]. The overall prevalence of inadequate GWG in Africa is not well understood. Comprehensive figures are not abundant, but a study conducted using the Demographic and Health Survey (DHS) data indicated that on average, a pregnant woman in sub‐Saharan Africa gains 6.6 kg, which was considerably low as compared with the corresponding estimates for Latin America, Caribbean, Europe, and Central Asia, having average GWG ranging from 11.80 to 11.19 kg [1, 5, 6]. Hence, more than 58% of pregnant women, particularly underweight ones in low‐income sub‐Saharan countries, experienced inadequate GWG [1, 7, 8]. In Ethiopia, although data on inadequate GWG are scarce, a few studies revealed that the prevalence ranges from 21.53% to 69.3% [9–11].

Inadequate GWG is a well‐recognized risk factor for poor pregnancy outcomes. It is highly associated with the development of a small for gestational age fetus, prematurity, and low birthweight, which subsequently may lead to prolonged hospital stay and high healthcare costs [3]. Recent studies have also found that low birthweight increases the risk for future development of noncommunicable diseases such as diabetes and cardiovascular disease in later life. On the other hand, preterm birth leads to loss of months of fetal development, leaving the infant vulnerable to morbidities, many of which are unique to the preterm population. As a result, every year, 1.1 million babies die from complications of preterm birth [12–14]. Because it results in these very serious consequences, inadequate GWG continues to be a significant public health problem globally [1, 4].

There are multitudes of factors that increase the risk for the occurrence of inadequate GWG. These include health system–related factors and maternal‐related factors, including socioeconomic, physiological, psychosocial, and behavioral factors [4, 5, 15]. Infrequent ANC visits, high maternal age, maternal low educational status, smoking, alcohol use, insufficient dietary intake, and lack of knowledge about appropriate foods to take during pregnancy are some of the risk factors that determine the occurrence of inadequate GWG [3, 5, 11, 16].

In response to the current WHO recommendation of increasing antenatal care (ANC) visits from four to eight contacts, which creates a good opportunity for weight gain monitoring and counseling interventions as a standard component of ANC practice, the guideline recommends the utilization of weight monitoring charts in each contact and providing counseling to improve diets and nutrient intake during pregnancy, along with physical activity to gain an adequate amount of weight and a positive pregnancy outcome [17, 18]. Ethiopia has developed a guideline to improve maternal and fetal health outcomes, adapting the eight contacts ANC by 2022. However, the country lacks appropriate guidelines and tools for monitoring GWG and providing appropriate counseling, support, and advice [19]. The IOM GWG recommendations, primarily based on Caucasian and black women in the United States, may not be suitable for low‐income settings like Ethiopia, suggesting Ethiopia should develop its own guidelines or use IOM guidelines cautiously [1, 9, 10].

There is limited evidence focusing on inadequate pregnancy weight gain in Ethiopia, and none in the study settings. On top of this, most of the studies did not properly assess variables like physical activity using the proper tool, which this study will address by employing the standard Global Physical Activity Questionnaire (GPAQ) tool. This actively demonstrates that knowing and understanding the problem in a local context is very important to intervene accordingly. Therefore, this study is aimed at assessing inadequate GWG and its associated factors among pregnant women in Gamo zone public hospitals.

## 2. Objectives

• We are aimed at assessing inadequate GWG among pregnant women in Gamo zone public hospitals, South Ethiopia, 2024.
• We are aimed at identifying the factors associated with inadequate GWG among pregnant women in Gamo zone public hospitals, South Ethiopia, 2024.

## 3. Methods

### 3.1. Study Design, Period, and Settings

A facility‐based cross‐sectional study was conducted from February 01, 2024, to March 30, 2024. The study was conducted in public hospitals of the Gamo zone, South Ethiopia. Arba Minch is the administrative center of the Gamo zone, which is located 443 km away from Addis Ababa, the capital city of Ethiopia, due south on the way to Butagira and 275‐km southwest. There is one general hospital, five primary hospitals, 58 health centers, and 303 health posts that provide maternal and child health services in the zone. Based on data from the Gamo zone health department, the estimated number of women who are in childbearing age group is 376,413, of which 55,897 are pregnant women who have ANC follow‐up in all health facilities annually. According to the data, 1323 pregnant women received ANC at least once [20].

### 3.2. The Study Population

All pregnant women who attended ANC first at ≤ 12 weeks of gestation and who came for the last ANC contact in Gamo zone public hospitals were the source population. Whereas all randomly selected pregnant women who attended ANC first at ≤ 12 weeks of gestation and who came for the last ANC contact in Gamo zone public hospitals were the study population. All pregnant women who attended ANC first at ≤ 12 weeks of gestation and who came for their last ANC contact in Gamo zone public hospitals were included in this study. Pregnant women diagnosed as having twin or above pregnancy, poly or oligohydramnios, and information about their weight is missing in the first visit, women diagnosed as having a uterine tumor, myoma, and pregnancy at the same time were excluded from the study.

### 3.3. Sample Size Determination and Sampling Procedure

**Figure 1:**
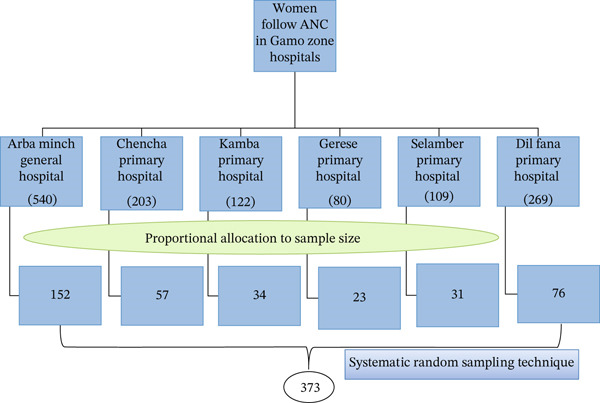
Schematic representation of sampling procedure to assess inadequate gestational weight gain and associated factors among pregnant women in Gamo Zone Public Hospitals, South Ethiopia, 2024.

The sample size was determined using a single population proportion formula. The following assumptions were considered, and 10% of the sample was added by considering possible nonresponse during the actual data collection process. *n* = (*Z* *α*/2)^2^∗*P*(1 − *p*)/*d* ^2^, where *n* is the sample size, *p* is the proportion of inadequate GWG taken as 67.2% from the previous study [9], Z_*α*/2_ = 1.96 at a confidence level of 95%, and *d* is the margin of error (5%). Therefore, the sample size was calculated to be 339, and after adding a 10% nonresponse rate, the final sample size was 373 used to conduct the study. There are six hospitals that are providing maternal and child healthcare services in the Gamo zone, and all hospitals were included in this study. Based on last year′s report, the number of women who attended the last ANC contact in each hospital was identified. Hence, in Arba Minch general hospital, 540 women were identified, Chencha primary hospital, 203, Kamba primary hospital, 122, Gerese primary hospital, 80, Selamber primary hospital, 109, and Dilfana hospital, 269. Then, the calculated sample size was proportionally allocated to each hospital. Finally, a systematic random sampling technique was applied to select the study participants; *K* values were calculated for each hospital and approximated as 5 (Figure 1).

### 3.4. Data Collection Instrument, Personnel, and Procedures

Primary data were collected by using an interviewer‐administered and pretested questionnaire, anthropometric measurements, and secondary data by reviewing the medical records of the clients. The questionnaire contains items on sociodemographic and economic characteristics of women, reproductive and medical factors, behavioral characteristics of women, and diet‐related factors of women. The questionnaire was uploaded to Open Data Kit (ODK).

The minimum dietary diversity of study participants was determined by employing multiple‐pass 24‐h recall method. First women were asked about foods consumed during the previous 24 h, then the interviewers were asked for foods possibly forgotten. They were asked about the time and occasion of each food item consumed. A detailed description of the amount and additions for each consumed food was also requested. Finally, the interviewer requested them to remember if anything else was consumed. Then the listed food items were categorized into 10 food groups, which were adopted from Food and Nutrition Technical Assistance (FANTA) III [21]. The wealth index of the households was measured by asking questions about their household assets [22]. The level of physical activity was measured using the GPAQ, which focuses on activity at work, during transport, and recreational activities [23].

Food security status was assessed using a complete form of the Household Food Insecurity Access Scale (HFIAS), which was developed and validated by FANTA. HFIAS has nine‐item questions with three domains of food insecurity (anxiety/uncertainty about the household food supply, insufficient quality of food, and insufficient food intake). The questions were categorized as “yes” or “no”, which represent occurrence, and under each “yes” category, there were three options of occurrence frequency (*r* *a* *r* *e* *l* *y* = 1, *s* *o* *m* *e* *t* *i* *m* *e* *s* = 2, and *o* *f* *t* *e* *n* = 3) [24].

Six data collectors who had a diploma in midwifery participated in the data collection process, and six health officers were assigned as supervisors.

### 3.5. Measurements and Anthropometry

Gestational age was estimated by the last menstrual period [9]. GWG was calculated by subtracting the initial weight from the total weight gained at the end of pregnancy [25]. Midupper arm circumference (MUAC) and the weight of women were measured by using calibrated and standardized equipment.

### 3.6. Study Variables

The dependent variable was inadequate GWG. The independent variables include sociodemographic and economic variables: age, educational status, wealth index, and occupational status; behavioral characteristics: physical activity level, smoking, alcohol consumption; diet‐related factors: dietary diversity score, nutritional status, food security status, and dietary habit; and obstetric and medical factors: number of gravidity and parity, gestational age, ANC contacts, chronic medical illness, and acute medical illness.

### 3.7. Operational Definition

Inadequate GWG occurs if a woman gains less than 10 kg of weight at the end of pregnancy [19, 26]. Initial weight gain is a weight measured at first ANC contact, and total weight gain is that measured during the last ANC contact [25]. Dietary diversity was measured using FANTA III, and the local food items were categorized into 10 food groups [21]. Women′s dietary diversity score was calculated by summing up the intake of the food groups over a period of 24 h before the interview, and categorized based on the mean score. Those who scored above the mean value were considered to have a good dietary diversity score, and below the mean value as a poor dietary diversity score.

Vigorous‐intensity activity means activity that makes a woman breathe much harder than normal, like forestry (cutting, chopping, and carrying wood), intense farm work (plowing, manual tilling of soil, and cutting crops), grinding with pestles or stones, and laboring [23]. Moderate‐intensity activity means activity that makes a woman breathe somewhat harder than normal like brisk walking; moderate farm work like domestic chores (cleaning, washing, cooking, milking cows, and drawing water); moderate farm work (gardening, planting, weeding, harvesting crops, and digging dry soil), weaving, tending animals, walking with load on head, wood work, and general building tasks (roofing, painting) [23]. Physical activity level was measured using the total time spent in physical activity during a typical week, and the intensity of the physical activity metabolic equivalent (MET) minutes was calculated. Those women who achieved 600 or more MET‐minutes per week were considered as physically active women, and less than 600 MET‐minutes per week were considered as physically inactive women [23].

### 3.8. Data Quality Management

The questionnaire was translated into Amharic and back to English for consistency. A pretest was carried out on 5% of the study sample at Humbo Primary Hospital. All the necessary corrections will be made based on the pretest result to avoid any confusion and for better completion of the questions. To assure data quality, training was given for data collectors and supervisors for 1 day on data collection method, data collection software (ODK), and anthropometric measurements by the principal investigator. To test the ability of data collectors on anthropometric measurements, a standardization test was done. To check standardization, each data collector measures sample clients two times and records their results to compare with the principal investigator′s measurement record as a reference. The technical error of measurement was calculated by using Ena Smart software. If any data collector failed to pass the standardization procedure, additional training was given accordingly. During data collection, the data collector took two separate MUAC measurements and weight, and the average value was calculated for reporting. The calibration of the weighing scale was checked before every measurement. The overall data collection process was supervised by supervisors and investigators. The current study was reported by following the Strengthening the Reporting of Observational Studies in Epidemiology (STROBE) checklist.

### 3.9. Data Processing and Analysis

The collected data was downloaded from the ODK aggregate as a CSV file and exported to Statistical Package for the Social Sciences (SPSS) Version 25.0 statistical software for further analysis. Exploratory data analysis was done to check the presence of potential outliers and normality (by the skewness and kurtosis test). Descriptive statistics such as simple frequencies, mean, and standard deviation were used to describe the sociodemographic and economic characteristics of participants as well as obstetric factors, medical illness, behavioral factors, and diet‐related factors. The household wealth index was constructed by using principal component analysis (PCA) after checking assumptions based on household assets, which are taken from the demographic health survey (DHS). This index is divided into three categories (tertiles), and each household was assigned to one of these categories of household wealth index (low, medium, and high). Based on the Household Food Insecurity Access Prevalence determination, households were categorized into four categories (food secure, mildly, moderately, and severely food insecure). The latter three groups were further categorized as food insecure. To identify factors associated with inadequate GWG, a binary logistic regression model was fitted. A bivariable analysis was used to identify candidate variables for multivariable analysis by using a crude odds ratio along with 95% CI. All variables with a significant association in bivariable analysis at *p* value < 0.25 were entered into a multivariable logistic regression model to assess the adjusted association between dependent and independent variables. A multivariable logistic regression model to control for potential confounders was performed with the backward elimination likelihood method to identify factors associated with inadequate GWG. Statistical significance was defined at a *p* value < 0.05 in the final model. An adjusted odds ratio (AOR) along with a 95% confidence interval was used to assess the strength of association. Hosmer–Lemeshow goodness of fit statistic was used to check model fitness and was satisfied (prob > chi2 = 0.195). The pseudo R^2^ of the final model was found to be 0.102. Multicollinearity was checked by using the variance inflation factor (VIF) and tolerance, with an average VIF of 1.252, showing no threat of multicollinearity. Finally, the result of the study was presented by using text, tables, and figures.

### 3.10. Ethical Approval

Before data collection, ethical clearance and approval were obtained from the institutional review board of Arba Minch College of Health Sciences with a reference number (AMCHS/01/10/3310), and an official support letter was received from the college′s Research and Community Service Directorate. The cooperation letter was submitted to health facilities, and permission was obtained to get full access to the information from the respondents and their medical records. Informed written consents were obtained from the parents/guardians for pregnant women aged less than 18 years, and assent was obtained from the participant before the interview. Participants aged 18–49 years old were asked to provide written consent. Clear information was given to participants about the purpose and procedure of the study, the importance of their participation, the right to withdraw at any time if they want, privacy and confidentiality of the information given by each respondent. Personal identifiers like the name of the respondent were not included in the data collection. All the study procedures follow the Helsinki Declaration.

## 4. Results

### 4.1. Sociodemographic and Economic Characteristics of Pregnant Women

A total of 372 pregnant women participated in the study, with a response rate of 99.7%. The mean (SD) age of the respondents was 27.67 (5.9) years, and the minimum and maximum ages of the respondents were 16 and 42, respectively. Almost all of the respondents were married (95.2%). More than one fourth (27.1%) of the respondents had formal education, and about (33.8%) were housewives. Nearly half (45.3%) of respondents were from the medium socioeconomic class (Table 1).

**Table 1:** Sociodemographic and economic characteristics of pregnant women in Gamo zone, South Etiopia, 2024.

Variables	Categories	Frequency (372)	Percent (%)
Age	< 20	46	12.3
	20–29	185	49.6
	30–39	128	34.3
	≥ 40	13	3.5

Marital status	Never married	7	1.9
	Married	355	95.2
	Previously married	10	2.7

Educational status	Unable to read and write	71	19.0
	Primary	87	23.3
	High school	120	32.3
	College and above	94	25.2

Occupational status	Government employee	94	25.3
	Housewife	126	33.9
	Merchant	34	9.1
	Student	57	15.3
	Other	61	16.4

House wealth index	Low	122	32.7
	Medium	169	45.4
	High	81	21.7

### 4.2. Obstetric and Health‐Related Characteristics of Pregnant Women

The majority of respondents (70.2%) have four or more ANC contacts in their current pregnancy. Of them, 80.7% have started ANC in eight or above 8 weeks of gestation. Half of the women (48.3%) got pregnant for the first time. Among 193 women who were pregnant more than once, 47.5% had ANC follow‐up during their last pregnancy. Among women who gave birth, 25.7% of the respondents have three or more children. Thirty‐five (9.4%) of pregnant women had an illness in the past 15 days. Of those, 2.4% have urinary tract infection, 1.9% have hypertension, and 5.1% have experienced headache, cold, and gastritis.

### 4.3. Diet‐Related, Environmental, and Behavioral Characteristics and Nutritional Status of Pregnant Women

**Figure 2:**
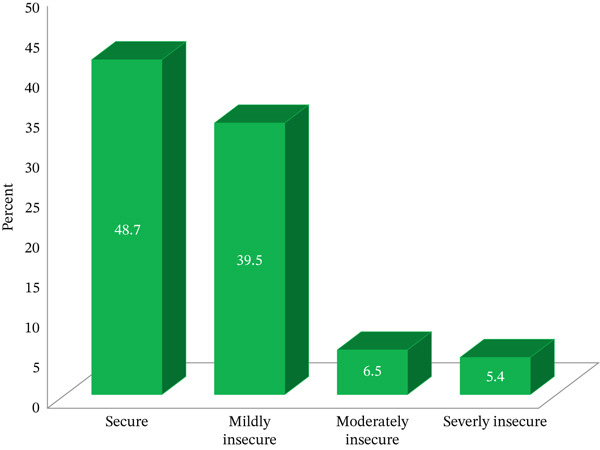
Food security status of pregnant women in Gamo zone, South Ethiopia, 2024.

The mean (SD) dietary diversity score of the respondents was 2.98 (1.01). Two hundred seventy‐eight (74.7%) and 191 (51.3%) of pregnant women had poor dietary diversity scores and from households experienced food insecurity, respectively (Figure 2). The majority (86.9%) of the respondents typically eat a meal 3–4 times a day, and more than half (56.8%) of the respondents take an additional snack (Table 2). Concerning the nutritional status of pregnant women, 123 (33%) of them have acute malnutrition. Almost all of the respondents (99.3%) were from households that had a latrine; however, only 46 (12.4%) of them had used an improved latrine. Forty‐nine respondents (13.1%) used an unimproved water source, and 29.2% treat water. About 27.1% of the respondents reported that home gardening was available in their homes. Fifty‐four respondents (14.5%) produce fruit and vegetables in their home gardens. Among them, 19.3% of them cultivated in the home garden only for home consumption purposes.

**Table 2:** Dietary practice and behavioral characteristics of pregnant women in Gamo zone, South Ethiopia, 2024.

Variables	Categories	Frequency	Percent
Meal consumption per day	< 2 times	41	11
	3–4 times	324	86.9
	≥ 5 times	8	2.1

Habit of having snacks in a day	Yes	212	56.8
	No	160	42.9

Habit of skipping breakfast within a week	Never	325	87.1
	1– 4 days	44	11.8
	≥ 5 days	4	1.1

Habit of eating away from home in a week	Never	301	80.7
	1–4 days	71	19
	≥ 5 days	1	0.3

Habit of eating fast food in a week nutritional status (MUAC)	Never	232	62.2
	1–4 days	137	36.7
	≥ 5 days	4	1.1
	< 23 cm	123	33
	≥ 23 cm	249	66.9

Alcohol drinking status	Yes	31	8.3
	No	341	91.4

Smoking status	Yes	3	0.8
	No	369	98.9

Physical activity status	≥ 600 MET	319	85.7
	< 600MET	53	14.3

Regarding the behavioral characteristics of the respondent, nearly all of the participants (98.9%) did not smoke cigarettes. Only 31 (8.3%) of pregnant women have a history or recent intake of alcohol. According to the WHO recommendation on physical activity, 319 (85.7%) women spent 600 or more MET‐minutes per week. Two hundred thirty‐nine (56.6%) and 122 (28.9%) of women spent less than 75 min in vigorous intensity activity and 150 min in moderate intensity activity throughout the week, respectively. (Table 2).

### 4.4. Prevalence of Inadequate GWG

In general, the prevalence of inadequate GWG among pregnant women in Gamo zone public facilities was 53.1% (95% CI: 48%, 58.4%). Also, 28.7% of pregnant women gained weight within the average value of the WHO recommendation, and 18% of women gained above the recommendations.

### 4.5. Factors Associated With Inadequate GWG

In this study, the following variables were entered into bivariable analysis to identify candidate variables for multivariable analysis, which include gestational age at first ANC contact, women′s educational status, gravidity, history of illness in the past 15 days, nutritional status of pregnant women, food security status, age, occupation, and frequency of ANC contact. From those variables with a *p* value < 0.25 were candidate variables for multivariable binary logistic regression. These include gestational age at first ANC contact, women′s educational status, gravidity, history of illness in the past 15 days, and frequency of ANC contact. In multivariable binary logistic regression, gestational age at first ANC contact, women′s educational status, and gravidity were significantly associated with inadequate GWG among pregnant women. Unlike pregnant women who started ANC contacts in less than 8 weeks, pregnant women who started ANC contact after 8 weeks were 1.79 times more likely to gain inadequate GWG (AOR = 1.79; CI: 1.02, 3.14). The odds of inadequate GWG were 2.85 times higher in pregnant women who are unable to read and write as compared with pregnant women who have a higher educational status (AOR = 2.86; CI: 1.37, 5.98). Primigarvida women were 1.673 times more at risk than that of multigravida women (AOR = 1.67; CI: 1.07, 2.62) (Table 3).

**Table 3:** Factors associated with inadequate gestational weight gain among pregnant women in Gamo zone public hospitals South Ethiopia, 2024.

Variables	Categories	Inadequate weight gain: *N* (%)	Normal and above normal: *N* (%)	COR (95% CI)	AOR (95% CI)	*p* value
Gestational age at first ANC contacts	Less than 8	45 (12.1)	26 (7.0)	1	1	
	8–12	153 (41.1)	148 (39.8)	1.67 (0.35, 1.02)	1.79 (1.02, 3.14)	0.042 ^∗^

Women′s education	Unable to read and write	50 (13.4)	21 (5.6)	2.10 (1.09, 4.02)	2.86 (1.37, 5.98)	0.005 ^∗^
	Primary	36 (9.7)	51 (13.7)	0.62 (0.35, 1.12)	0.74 (0.39, 1.44)	0.379
	Secondary	62 (16.7)	58 (15.6)	0.94 (0.55, 1.62)	1.11 (0.62, 2.00)	0.724
	College and above	50 (13.4)	44 (11.8)	1	1	

Gravidity	Primigravida	105 (28.2)	75 (20.2)	1.49 (0.99, 2.25)	1.67 (1.07, 2.62)	0.02 ^∗^
	Multigravida	93 (25.0)	99 (26.6)	1	1	

Frequency of ANC contacts	< 4 times	62 (16.7)	45 (12.1)	1.31 (0.49, 1.20)	1.08 (0.65, 1.80)	0.764
	≥ 4 times	136 (36.6)	129 (34.7)	1	1	

History of illness	Yes	23 (66.7)	12 (34.3)	1.77 (0.86, 3.68)	1.70 (0.79, 3.64)	0.177
	No	175 (51.9)	162 (48.1)	1	1	

^∗^Statistically significant at *P*‐value < 0.05.

## 5. Discussion

The current study is aimed at assessing the magnitude of inadequate GWG and associated factors. Hence, it was identified that the magnitude of insufficient GWG was 53.1% (95% CI: 48%, 58.4%), and the first ANC visit after 8 weeks of gestational age, inability to read and write, and primigravidity were identified as the significant predictors of inadequate GWG.

Adequate GWG is essential for optimal pregnancy outcomes. However, in this study, more than half (53.1%) of the pregnant women experienced inadequate GWG. The findings from the current study were in line with a prospective cohort study done in Japan, which reported a 52.6% prevalence of inadequate GWG [27]. It was in line with a meta‐analysis conducted in low‐ and middle‐income countries that reported a prevalence of inadequate GWG of 54% [16].

This result is higher than the global pooled prevalence of inadequate GWG, which was reported to be 39.4%, based on the IOM guidelines using systematic review and meta‐analysis [4]. It was also higher than the finding from a prospective cohort study done in Gurage zone, Central Ethiopia, that reported 26% inadequate GWG [28]. The difference in the reported rates of inadequate weight gain between this study and the systematic review may be due to two key factors. First, differences in the guidelines used to classify GWG. In this study, we utilized the ANC guidelines compiled by the Ministry of Health of Ethiopia as per the WHO ANC recommendations, rather than the IOM guidelines used in the systematic review. The use of different classification criteria can lead to variations in the reported prevalence of inadequate weight gain. The other key factor might be due to population‐level differences. The study population in this research may have unique characteristics that contribute to a higher rate of inadequate GWG compared with the global prevalence. Factors such as socioeconomic status, access to healthcare, or cultural practices can influence weight gain patterns during pregnancy. The discrepancy between the local study findings and the global trend highlights the importance of considering regional and population‐specific factors when examining GWG outcomes. Utilizing appropriate guidelines and understanding the local context are crucial for accurately assessing and addressing the issue of inadequate weight gain during pregnancy. Moreover, differences in study design might also contribute to the discrepancy since the study done in Japan and the Gurage zone, Ethiopia, utilized a prospective study design.

This prevalence is also higher than from the results reported from the studies conducted in Korea (30.3%), China (14.7%), Cameroon (14.7%), Tanzania (42%), and Malaysia (32%). The significantly higher rate of inadequate weight gain observed in this study, compared with these other settings, suggests that there may be unique factors. These might be due to the differences in socioeconomic and demographic characteristics; this population has a higher proportion of women with a lower rate of formal education and other risk factors that predispose them to inadequate weight gain during pregnancy. In addition to this, cultural and dietary influences might have an effect. Local dietary patterns, food availability, and cultural norms around pregnancy weight gain could play a role in the observed higher prevalence in this study population [29–33].

Contrarily, the prevalence of inadequate GWG reported in this study is actually lower than the results from previous studies conducted in Addis Ababa (67%) [9], Harrar (69.3%) [10], and a bidirectional cohort study conducted in West Shoa, Ethiopia [34]. This difference might be attributed to the use of an updated ANC guideline in the current study. The updated ANC guideline, which increased the recommended number of ANC contacts from 4 to 8, may have contributed to the lower rate of inadequate weight gain observed in this study population. The additional ANC visits and closer monitoring of pregnant women could provide more opportunities for healthcare providers to closely track and support women′s GWG and allow for more tailored counseling and interventions to help women achieve the recommended weight gain targets during pregnancy. In addition to these, the higher rates of inadequate weight gain observed in Addis Ababa and Harrar might be due to region‐specific factors within Ethiopia that contribute to this issue. It might also be because of a difference in study design.

The study found that mothers who had not attended formal education were 2.7 times more likely to experience inadequate GWG compared with their educated counterparts. This aligns with findings from a related study conducted in Addis Ababa [9]. It also showed that maternal education level is a significant factor associated with GWG. Lower educational attainment appears to increase the risk of insufficient weight gain during pregnancy. The connection between maternal education and adequate GWG highlights the importance of promoting access to education, especially for women. Improved education may empower mothers with the knowledge and resources to achieve optimal weight gain, which is crucial for healthy pregnancy outcomes. Conversely, lower maternal education may limit awareness of recommended gestational nutrition, food choices, or the importance of weight gain, leading to inadequate caloric or protein intake, poor dietary diversity, or meal frequency, further resulting in inadequate GWG. In addition, education might influence women′s empowerment, which in turn affects their ability to make decisions regarding diet, workload, and healthcare seeking, and higher GWG was associated with higher women′s empowerment.

The findings of this study highlight the statistically significant association between gestational age at first ANC contacts and inadequate GWG among pregnant women. The results show that women who initiated ANC after 8–12 weeks of gestation were 1.79 times more likely to experience inadequate weight gain during pregnancy compared with those who started care earlier. Timely initiation of ANC is crucial for the proper monitoring and management of a woman′s health and weight during pregnancy. Late enrollment in antenatal services may limit the opportunities for healthcare providers to intervene and counsel women on appropriate weight gain, nutrition, and lifestyle factors that support healthy fetal development. There are no previous studies that examine gestational age at first ANC contact as an important predictor of inadequate weight gain during pregnancy, but the WHO recommends that it is crucial to have at least one ANC contact below 12 weeks of gestation to improve maternal and child health [17]. Possible explanations for the association between gestational age at first ANC contact and inadequate weight gain include reduced opportunities for nutrition counseling, missed chances for early identification and management of pregnancy‐related complications, and limited time for healthcare providers to implement appropriate weight gain monitoring and support strategies. First‐time mothers often encounter societal restrictions, especially regarding food intake and rest, due to concerns about childbirth complications and resource limitations. Lacking prior experience, they may feel insufficiently confident to challenge these norms, which further results in inadequate GWG.

The findings of this study reveal a statistically significant association between primigravida (first‐time pregnancy) and an increased risk of inadequate GWG among pregnant women. The results indicate that first‐time mothers were 1.67 times more likely to experience inadequate weight gain during pregnancy compared with women with previous pregnancies. The result is in line with the study conducted in Gondar [11]. This might be due to primigravida women having limited exposure to healthcare provider guidance and education on appropriate weight gain during pregnancy. First‐time mothers tend to rely more heavily on advice from family or peers on not “eating too much” to avoid a “big baby” and beliefs that weight gain leads to difficult or “unnatural” labor because of these mothers′ strain from feeding on a nutritious diet and being affected by inadequate weight gain.

The current study evidenced that more than half of the pregnant women who are unable to read and write had inadequate GWG, which increased the likelihood of the problem by nearly three times. This is consistent with the factors of primigravity and late ANC booking after 8 weeks of conception, which increased the risk by nearly two times for both. The finding is particularly relevant for similar settings because the factors identified in the study setting might be closely similar to contextual and structural challenges common across such settings. High levels of women′s illiteracy are widespread in many of the low‐income settings, particularly rural areas. Limited literacy hinders women′s ability to understand, access, and act on nutrition and pregnancy‐related information, making inadequate GWG more likely. Also, primigravidity is particularly relevant in rural settings where primigravida women may have limited pregnancy and nutrition‐related knowledge. Moreover, late ANC booking typically echoes systemic barriers of rural health systems in resource‐limited settings, like lack of transportation cost, far distances to health facilities, shortages of skilled healthcare providers, and competing household responsibilities.

Emphasis was given on intensive training, pretesting, and active field data collection supervision to minimize bias. Since the study participants were women who attended the public hospitals in the zone, the findings will not represent those outside of these public hospitals. As a limitation, it might be difficult to establish a temporal relationship because of the nature of the study design. Even if we are giving enough time to remember what they did or the data collectors were probing the respondents, we may introduce recall bias when measuring household food security, physical activities, and dietary diversity. Social desirability bias was also a limitation when collecting data regarding dietary diversity, alcohol consumption, and cigarette smoking since the data collectors were healthcare providers.

### 5.1. Policy and Program Implication

The pregnancy period is the critical time in the human life cycle for implementing interventions to ensure life‐long maternal and child health. However, there is a lack of evidence‐based standardized public health tools that are applicable to all women from diverse geographical locations, which help to monitor GWG. The IOM guidelines were developed based on the recommendations from findings of observational studies from high‐income countries. However, the aforementioned does not work for low‐income countries, and this initiated the WHO to develop global weight gain standards that can be used as a tool for dynamic monitoring in ANC in diverse settings [35]. Despite this recommendation, the current study indicated that the prevalence of inadequate gestational weight was highly predicted by late ANC booking, unable to read and write, and primigravidity. The findings of the current study are important for policy, program, and practice in the zone. Hence, the finding highlights that policymakers and programmers should focus on ensuring optimal GWG to attain SDG 3 through the development and implementation of context‐specific ANC guidelines that help early ANC booking and designing tailored interventions. Furthermore, this important public health problem needs integrated, tailored, and consistent interventions from the zone, Regional Health Bureau, partners, and other stakeholders to reduce maternal and child mortality. Likewise, designing health strategies and policies should take into account encouraging women′s education and ensuring early ANC visits, which are contributors to inadequate GWG.

## 6. Conclusion

The findings of this study revealed that more than half of pregnant women still gain inadequate weight, and it has complex relationships with various factors, including women′s education, primigravidity, and gestational age at first ANC contact. Strengthening early ANC booking, enhancing nutrition counseling tailored to primigravidas, and developing policies that address educational and sociocultural barriers could contribute to more effective ANC guidelines and improved maternal nutrition outcomes in similar settings. In addition, to effectively address the issue of inadequate GWG, a comprehensive approach that considers the interplay of these various factors is essential. Healthcare providers, policymakers, and community stakeholders must work collaboratively to develop and implement multifaceted interventions that target the specific needs of women with lower education, primigravida women, and those who delay their first ANC contact. By addressing these key determinants, public health efforts can strive to promote optimal GWG and improve maternal and child health outcomes. Moreover, since this study did not assess the calorie intake of pregnant women, which has a great impact on GWG, further studies were highly encouraged by considering dietary intake using a prospective method of dietary assessment (weighted food record and estimated food record).

## Author Contributions

S.W.K. and T.G.G. conducted the research. R.H., S.T., and R.A.A. supervised data collection. S.W.K., F.M., and T.G.G. completed the statistical analyses and drafted the manuscript. S.W.K. and T.G.G. contributed to the writing of the manuscript. All authors contributed to the article. S.W.K. and T.G.G. contributed equally to this work.

## Funding

No funding was received for this manuscript.

## Disclosure

S.W.K. and T.G.G. were responsible for the design of the study. All authors approved the submitted version.

## Conflicts of Interest

The authors declare no conflicts of interest.

## Data Availability

The datasets used and/or analyzed during the current study are available from the corresponding author upon reasonable request.

## References

[1] Kac G. , Carrillo T. , Rasmussen K. , and Del Rosso J. , What We Know About Weight Gain During Pregnancy in Low and Middle Income Countries, 2022, Alive & Thrive, Technical brief https://www.aliveandthrive.org/sites/default/files/technical_brief_gestational_weight_gain_v15mdcit.pdf.

[2] World Health Organization Technical Report , The Use and Interpretation of Anthropometry, 1995, World Health Organization.

[3] Rasmussen K. M. and Yaktine A. L. , Weight Gain During Pregnancy: Reexamining the Guidelines, 2009, National Academies Press. 20669500

[4] Martinez-Hortelano J. A. , Cavero-Redondo I. , Alvarez-Bueno C. , Garrido-Miguel M. , Soriano-Cano A. , and Martinez-Vizcaino V. , Monitoring Gestational Weight Gain and Prepregnancy BMI using the 2009 IOM Guidelines in the Global Population: A Systematic Review and Meta-Analysis, BMC Pregnancy and Childbirth. (2020) 20, no. 1, 10.1186/s12884-020-03335-7, 33109112. PMC7590483 33109112

[5] Gebremedhin S. and Bekele T. , Gestational Weight Gain in Sub-Saharan Africa: Estimation Based on Pseudo-Cohort Design, PLoS One. (2021) 16, no. 5, e0252247, 10.1371/journal.pone.0252247, 34038488. 34038488 PMC8153429

[6] Wang D. , Wang M. , Darling A. M. , Perumal N. , Liu E. , Danaei G. , and Fawzi W. W. , Gestational Weight Gain in Low-Income and Middle-Income Countries: A Modelling Analysis Using Nationally Representative Data, Health. (2020) 5, no. 11, 10.1136/bmjgh-2020-003423. PMC7661366 33177038

[7] Asefa F. , Cummins A. , Dessie Y. , Hayen A. , and Foureur M. , Gestational Weight Gain and Its Effect on Birth Outcomes in Sub-Saharan Africa: Systematic Review and Meta-Analysis, PLoS One. (2020) 15, no. 4, e0231889, 10.1371/journal.pone.0231889, 32324783. 32324783 PMC7179909

[8] Johnson J. L. , Farr S. L. , Dietz P. M. , Sharma A. J. , Barfield W. D. , and Robbins C. L. , Trends in Gestational Weight Gain: The Pregnancy Risk Assessment Monitoring System, 2000-2009, American Journal of Obstetrics and Gynecology. (2015) 212, no. 6, 806.e1–806.e8, 10.1016/j.ajog.2015.01.030, 2-s2.0-84931574978. PMC4629490 25637844

[9] Asefa F. , Cummins A. , Dessie Y. , Foureur M. , and Hayen A. , Patterns and Predictors of Gestational Weight Gain in Addis Ababa, Central Ethiopia: A Prospective Cohort Study, Reproductive Health.(2021) 18, no. 1, 10.1186/s12978-021-01202-y, 34321037. PMC8317358 34321037

[10] Asefa F. and Nemomsa D. , Gestational Weight Gain and Its Associated Factors in Harari Regional State: Institution Based Cross-Sectional Study, Eastern Ethiopia, Reproductive Health. (2016) 13, no. 1, 10.1186/s12978-016-0225-x, 2-s2.0-84984783704, 27576539. PMC5004260 27576539

[11] Engidaw M. T. , Gebremariam A. D. , Abate B. A. , Tesfa D. , Tiruneh S. A. , Addisu Y. , and Belachew Y. Y. , Magnitude and Factors Associated With Gestational Weight Gain Adequacy Among Pregnant Women in South Gondar Zone, Northwest Ethiopia, Current Developments in Nutrition.(2023) 7, no. 12, 102031, 10.1016/j.cdnut.2023.102031, 38162997. 38162997 PMC10756953

[12] Ahmed T. , Hossain M. , and Sanin K. I. , Global Burden of Maternal and Child Undernutrition and Micronutrient Deficiencies, Annals of Nutrition & Metabolism. (2013) 61, no. Suppl 1, 8–17, 10.1159/000345165, 2-s2.0-84879487482. 23343943

[13] Frederick I. O. , Williams M. A. , Sales A. E. , Martin D. P. , and Killien M. , Pre-Pregnancy Body Mass Index, Gestational Weight Gain, and Other Maternal Characteristics in Relation to Infant Birth Weight, Maternal and Child Health Journal.(2008) 12, no. 5, 557–567, 17713848, 10.1007/s10995-007-0276-2, 2-s2.0-53749086325. 17713848

[14] Patel R. M. , Short- and Long-Term Outcomes for Extremely Preterm Infants, American Journal of Perinatology. (2016) 33, no. 3, 318–328, 10.1055/s-0035-1571202, 2-s2.0-84959159025, 26799967. 26799967 PMC4760862

[15] Lertbunnaphong T. , Talungjit P. , and Titapant V. , Does Gestational Weight Gain in Normal Pre-Pregnancy BMI Pregnant Women Reflect Fetal Weight Gain?, Journal of the Medical Association of Thailand.(2012) 95, no. 7. 22919977

[16] Darling A. M. , Wang D. , Perumal N. , Liu E. , Wang M. , Ahmed T. , Christian P. , Dewey K. G. , Kac G. , Kennedy S. H. , Subramoney V. , Briggs B. , Fawzi W. W. , and Members of the GWG Pooling Project Consortium , Risk Factors for Inadequate and Excessive Gestational Weight Gain in 25 Low- and Middle-Income Countries: An Individual-Level Participant Meta-Analysis, PLoS Medicine.(2023) 20, no. 7, e1004236, 10.1371/journal.pmed.1004236, 37486938. 37486938 PMC10406332

[17] Organization WH , WHO Recommendations on Antenatal Care for a Positive Pregnancy Experience: Summary: Highlights and Key Messages From the World Health Organization′s 2016 Global Recommendations for Routine Antenatal Care, 2018, World Health Organization.

[18] Vogel J. P. , Habib N. A. , Souza J. P. , Gülmezoglu A. M. , Dowswell T. , Carroli G. , Baaqeel H. S. , Lumbiganon P. , Piaggio G. , and Oladapo O. T. , Antenatal Care Packages With Reduced Visits and Perinatal Mortality: A Secondary Analysis of the WHO Antenatal Care Trial, Reproductive Health.(2013) 10, no. 1, 10.1186/1742-4755-10-19, 2-s2.0-84876900366. PMC3637102 23577700

[19] Ministry of Health , Ethiopian National Antenatal Care Guideline, 2022, Available from: http://repository.iphce.org/bitstream/handle/123456789/1647/ANC-GUIDELINE_Feb-24-2022.pdf.

[20] Department Gzh , Annual HMIS Report of Gamo Zone Health Breaue, 2014.

[21] Arimond M. , Ballard T. J. , Deitchler M. , Kennedy G. , and Martin-Prével Y. , Minimum Dietary Diversity for Women: A Guide for Measurement, 2016, FAO.

[22] Rutstein S. O. , Steps to Constructing the New DHS Wealth Index, 2015, ICF International.

[23] Organization WH , Global Physical Activity Questionnaire (GPAQ) Analysis Guide, 2012, World Health Organization.

[24] Coates J. S. A. and Bilinsky P. , Household Food Insecurity Access Scale (HFIAS) for Measurement of Food Access: Indicator Guide, 2007, Food and Nutrition Technical Assistance.

[25] Committee on Nutritional Status During Pregnancy , Nutrition During Pregnancy: Part I: Weight Gain, Part II: Nutrient Supplements, 1990, National Academies Press. 25144018

[26] National Research Council , Weight Gain During Pregnancy: Reexamining the Guidelines, 2009, National Academies Press. 20669500

[27] Aoyama S. , Haruna M. , Yonezawa K. , Tahara-Sasagawa E. , Usui Y. , Ohori R. , Tanaka M. , Fujita M. , Matsuzaki M. , Suetsugu Y. , Sato Y. , and Takeuchi A. , Factors Associated With Inadequate Gestational Weight Gain: A Prospective Multicenter Cohort Study, Nursing & Health Sciences.(2025) 27, no. 1, e70047, 10.1111/nhs.70047, 39914995. 39914995 PMC11802277

[28] Beyene G. A. , Yunus M. A. , Deribew A. B. , and Kasahun A. W. , Gestational Weight Gain and Its Determinants Among Pregnant Women in Gurage Zone, Central Ethiopia: A Cohort Study, BMC Women′s Health.(2024) 24, no. 1, 10.1186/s12905-024-03223-8, 38937766. PMC11212422 38937766

[29] Choi H. , Lim J.-Y. , Lim N.-K. , Ryu H. M. , Kwak D. W. , Chung J. H. , Park H. J. , and Park H. Y. , Impact of Pre-Pregnancy Body Mass Index and Gestational Weight Gain on the Risk of Maternal and Infant Pregnancy Complications in Korean Women, International Journal of Obesity.(2022) 46, no. 1, 59–67, 10.1038/s41366-021-00946-8. 34489525 PMC8748202

[30] Edi M. , Chin Y. S. , Woon F. C. , Appannah G. , Lim P. Y. , and MICOS Research Group , Inadequate Gestational Weight Gain and Exposure to Second-Hand Smoke During Pregnancy Increase the Risk of Low Birth Weight: A Cross-Sectional Study Among Full-Term Infants, International Journal of Environmental Research and Public Health.(2021) 18, no. 3, 10.3390/ijerph18031068. PMC7907990 33530307

[31] Halle-Ekane G. , Nsom J. , Atashili J. , Palle J. , Nsagha D. , Nguefack C. , and Njotang P. N. , Outcome of Pregnancy in Patients With Excessive Gestational Weight Gain in Two District Hospitals in Douala, Cameroon, 2015, SM Journal of Gynecology and Obstetrics.

[32] Wen T. and Lv Y. , Inadequate Gestational Weight Gain and Adverse Pregnancy Outcomes Among Normal Weight Women in China, International Journal of Clinical and Experimental Medicine.(2015) 8, no. 2, 2881–2886, 25932249. 25932249 PMC4402896

[33] Yang J. , Wang M. , Tobias D. K. , Rich-Edwards J. W. , Darling A. M. , Abioye A. I. , Pembe A. B. , Madzorera I. , and Fawzi W. W. , Gestational Weight Gain During the Second and Third Trimesters and Adverse Pregnancy Outcomes, Results From a Prospective Pregnancy Cohort in Urban Tanzania, Reproductive Health. (2022) 19, no. 1, 10.1186/s12978-022-01441-7. PMC9204988 35710384

[34] Terfassa T. G. , Wirtu D. , and Egata G. , Gestational Weight Gain and Its Determinants Among Pregnant Women Attending Antenatal Care at West Shawa Hospitals, Oromia, Ethiopia, PLoS One. (2025) 20, no. 6, e0323725, 10.1371/journal.pone.0323725, 40489558. 40489558 PMC12148144

[35] Development of Global Gestational Weight Gain Standards [Internet], [cited December 9, 2025]. Available from: https://www.who.int/teams/nutrition-and-food-safety/development-of-global-gestational-weight-gain-standards.

